# Modulation of Helper T Cytokines in Thymus during Early Pregnancy in Ewes

**DOI:** 10.3390/ani9050245

**Published:** 2019-05-16

**Authors:** Leying Zhang, Zimo Zhao, Hao Mi, Baoliang Liu, Bin Wang, Ling Yang

**Affiliations:** Department of Animal Science, College of Life Sciences and Food Engineering, Hebei University of Engineering, Handan 056021, China; zhangly056000@126.com (L.Z.); zhaozm0320@126.com (Z.Z.); mi137604660@163.com (H.M.); lblyxm@126.com (B.L.); hdwangbin@126.com (B.W.)

**Keywords:** cytokine, pregnancy, sheep, thymus

## Abstract

**Simple Summary:**

The thymus is a main organ of the immune system. Immune tolerance exists in maternal immune system during pregnancy. Helper T (Th)1 and Th2 cytokines regulate the functions of immune system. We found that early pregnancy affected the production of Th1 and Th2 cytokines in maternal thymus in sheep, which may be beneficial for normal pregnancy.

**Abstract:**

There is an immune tolerance in maternal immune system during pregnancy, and thymus is a main organ of the immune system. Helper T (Th)1 and Th2 cytokines are involved in the regulation of immune system, but the modulation of Th cytokines in the thymus during early pregnancy is unclear in ewes. Thymuses were collected on day 16 of the estrous cycle, and on days 13, 16, and 25 of pregnancy in ewes. qRT-PCR, Western blot, and immunohistochemistry were used to analyze the expression of Th1 and Th2 cytokines in the thymuses. There was a peak in the expression of interferon-gamma (IFN-γ) on day 16 of pregnancy, an upregulation of tumor necrosis factor beta (TNF-β), and a sustained expression of interleukin-2 (IL-2) and IL-4. Furthermore, there was a peak in the expression of IL-6 on day 13 of pregnancy, no expression of IL-6 on day 16 of the estrous cycle and day 25 of pregnancy, and an upregulation of IL-5 and IL-10 in the thymuses during early pregnancy. The immunohistochemistry results revealed that the IFN-γ and IL-6 proteins were limited to the stromal cells, capillaries, and thymic corpuscles. In conclusion, early pregnancy influenced the production of Th1 and Th2 cytokines of maternal thymus in sheep.

## 1. Introduction

Helper T (Th) cytokines regulate the functions of the immune system, and Th1 and Th2 cells are defined by their cytokine secretion profiles [1]. Cytokines (interleukin-2 (IL-2), interferon-gamma (IFN-γ), and tumor necrosis factor beta (TNF-β)) produced by Th1 lymphocytes induce cell-mediated cytotoxicity and inflammatory responses. Th2 cells produce Th2 cytokines (IL-4, IL-5, IL-6, and IL-10) to induce antibody production and improve mast cell and eosinophil granulocyte differentiation and activation [2,3]. Th1 cytokines are customarily harmful to successful pregnancy, and high levels of Th1 cytokines are present in the abortion animals. However, Th2 cytokines are beneficial for humoral response, and a conspicuous Th2-bias exists in successful pregnancy [4,5]. There is a significant increase in Th1 cytokines in women, which is the potential immune cause for recurrent spontaneous abortions [6]. Our previous study finds that *IFN-γ* is downregulated, and Th2 cytokines are upregulated in the bovine peripheral blood mononuclear cells (PBMCs) during early pregnancy [7]. The expression of TNF-β and IL-2 is decreased, while the expression of IL-5 and IL-10 is increased in the ovine maternal lymph nodes during early pregnancy [8].

The thymus is a main organ of the immune system. It is the site where T cells mature, and is vital for adaptive immune response. The maternal thymus undergoes significant involution during pregnancy in mammals, and recovers to normal size after birth [9], which is mediated by hormones and is necessary for fetal survival [10]. The thymic stromal progesterone (P4) receptor (PGR) is essential for the successful pregnancy, and the early T lymphopoiesis is prevented via a PGR-dependent paracrine mechanism during pregnancy in mice, which is essential for normal fertility [11]. Our previous study finds that there are changes in interferon-stimulated gene 15 kDa protein, PGR, and P4-induced blocking factor (PIBF) in thymus during early pregnancy in ewes [12]. However, the regulation of Th cytokines in ovine thymus induced by early pregnancy is unclear. The effects of early pregnancy on regulation of Th cytokines in ovine thymus are mainly due to P4 and interferon-tau (IFNT). There are significantly higher concentrations of P4 in plasma on days 12 and 13, and lower concentrations of P4 on days 15 and 16 during the ovine estrous cycle [13]. IFNT (Protein X) and additional proteins secreted by the trophoblast of blastocyst in the uterus are present on days 14–21 of pregnancy in ewes [14]. There is no day 25 of nonpregnancy, because the average of estrous cycle is 17 days in sheep. Day 13 of the estrous cycle is almost similar to day 13 of pregnancy according to the above reasons. In the present experiments, the thymic tissues from nonpregnant and early pregnant ewes were sampled to study the regulation of Th1 and Th2 cytokines, which may be helpful to understand the thymic immune regulation during early pregnancy in ruminants.

## 2. Materials and Methods

### 2.1. Animals and Experimental Design

Small-tail Han ewes (17–19 months of age) with normal estrous cycles, were freed from drugs and other stress stimuli, and were housed at a farm in China. The experimental protocol was approved by the Hebei University of Engineering Animal Care and Use Committee (AEEI-16015), and humane animal care and handling procedures were followed throughout the experiment. The ewes were observed daily for estrous behavior in the presence of caudaepididyectomized rams, randomly divided into 4 groups. The ewes of pregnant groups were mated to intact rams after detection of sexual receptivity (day 0), but the ewes of cyclic group were not mated with intact rams (*n* = 6 for each group). The samples of thymus were collected from the ewes on day 16 of the estrous cycle, and on day 13, 16, and 25 of gestation as described previously with thymuses instead of lymph nodes [8].

### 2.2. RNA Extraction and qRT-PCR Assay

RNA extraction from frozen thymic tissues was performed using TRIzol (Invitrogen, Carlsbad, CA, USA), and a FastQuant RT kit (Tiangen Biotech Co., Ltd., Beijing, China) was used to synthesize total RNA following the manufacturer’s instructions. A Bio-Rad CFX96 real-time PCR system (Bio-Rad, Hercules, CA, USA) was used to perform qRT-PCR with a kit as described previously in lymph node [8]. The primer sequences of ovine Th cytokines and *GAPDH* are listed in Table 1. *GAPDH* was used as an internal reference for normalization of mRNA expression, and 2^−ΔΔCt^ analysis method was used to calculate the gene expression values [15]. The gene expression level on day 16 of nonpregnant group was set as control.

### 2.3. Western Blot Analysis

The total proteins were extracted, and the protein concentration was measured as described previously [8]. Total proteins (10 μg/lane) were separated by SDS-PAGE followed by transferring onto 0.22 μm PVDF membranes (Millipore, Bedford, MA, USA). Blot analysis was performed with an anti-IFN-γ antibody (Abcam, San Francisco, CA, USA, ab27919), an anti-TNF-β antibody (Santa Cruz Biotechnology, Inc., Santa Cruz, CA, USA, SC-28345), an anti-IL-2 antibody (Abcam, ab193807), an anti-IL-4 antibody (Bio-Techne, MAB2469), an anti-IL-5 antibody (Santa Cruz Biotechnology, SC-8433), an anti-IL-6 antibody (Abcam, ab193853), and an anti-IL-10 antibody (Santa Cruz Biotechnology, SC-32815) at a dilution of 1:1000 to analyze expression of Th1 and Th2 cytokines respectively. Target proteins were detected by a Pro-light HRP chemiluminescence detection reagent (Tiangen Biotech, Beijing, China). An anti-glyceraldehyde 3-phosphate dehydrogenase (GAPDH) antibody (Santa Cruz Biotechnology, sc-20357, 1:1000) was used for normalization of sample loading. The intensity of blots were semi-quantified by Quantity One V452 (Bio-Rad Laboratories), and the values were calculated using GAPDH as an internal reference.

### 2.4. Immunohistochemistry Analysis

Transverse pieces of thymic samples were fixed in 4% buffered paraformaldehyde over 24 h, and embedded in paraffin and sectioned. The sections were stained by hematoxylin and eosin (HE). The sections were treated as described previously with thymuses instead of lymph nodes [12]. Immunohistochemical localizations of IFN-γ and IL-6 proteins in the thymus were performed by the IFN-γ antibody (Abcam, ab27919, 1:100) and IL-6 antibody (Abcam, ab193853, 1:100), respectively. The negative controls were handled with antiserum-specific isotype in place of IFN-γ or IL-6 antibody. The antibody binding sites were analyzed and scored as described previously [16].

### 2.5. Statistical Analysis

The data for the expression values of Th1 and Th2 cytokines mRNA and proteins were analyzed using the Proc Mixed models of SAS (Version 9.1; SAS Institute, Cary, NC, USA) and the Duncan method, as described previously with thymuses instead of lymph nodes [8]. A *p*-value < 0.05 was considered significantly different.

## 3. Results

### 3.1. Expression Values of Th1 Cytokines in the Thymuses

**Figure 1:**
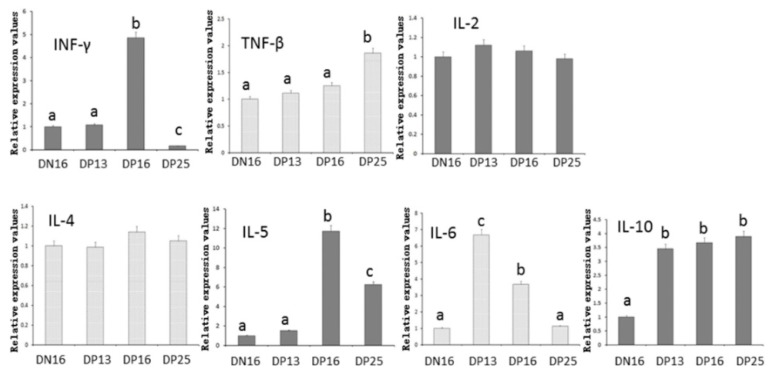
Relative expression levels of helper T cytokines mRNA in the thymuses measured by quantitative real time PCR. Note: DN16 = Day 16 of nonpregnancy; DP13 = Day 13 of gestation; DP16 = Day 16 of gestation; DP25 = Day 25 of gestation. Significant differences (*p* < 0.05) are indicated by different letters within same color column.

**Figure 2:**
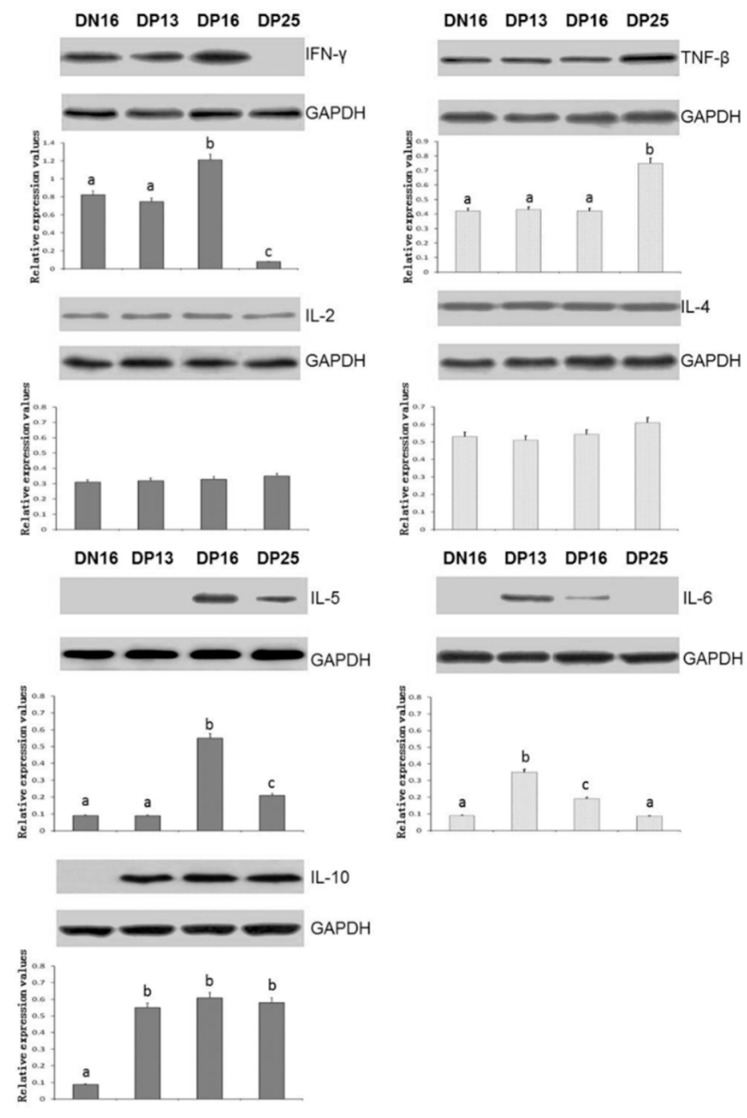
Expression of helper T cytokines proteins in the thymuses analyzed by Western blot. Note: DN16 = Day 16 of nonpregnancy; DP13 = Day 13 of gestation; DP16 = Day 16 of gestation; DP25 = Day 25 of gestation. Significant differences (*p* < 0.05) are indicated by different superscript letters within same color column.

It is shown in Figure 1 and Figure 2 that the expression of IFN-γ was upregulated on day 16 of gestation (*p* < 0.05), but the expression values were the lowest on day 25 of pregnancy among the four groups (*p* < 0.05). The expression of *TNF-β* mRNA and proteins increased on day 25 of gestation (*p* < 0.05), but early pregnancy did affect the expression of *TNF-β* mRNA and protein on days 13 and 16 of gestation (*p* > 0.05). Furthermore, early pregnancy had no effect on expression of *IL-2* mRNA and protein (*p* > 0.05).

### 3.2. Expression Values of Th2 cytokines in the Thymuses

Early pregnancy exerted no effect on the expression of IL-4 (*p* > 0.05) (Figure 1 and Figure 2). Expression of IL-5 reached a peak on day 16 of gestation, but expression values were lower in nonpregnancy and on day 13 of gestation in the thymuses (*p* < 0.05). The expression values of IL-6 upregulated on day 13 of gestation (*p* < 0.05), and declined from day 13 to 25 of gestation (*p* < 0.05) (Figure 1 and Figure 2). The expression values of IL-10 decreased during the estrous cycle (*p* < 0.05) (Figure 1 and Figure 2), but early pregnancy enhanced the expression of IL-10 in the thymuses (*p* < 0.05).

### 3.3. The Immunohistochemistry for IFN-γ and IL-6 Proteins in the Thymuses

**Figure 3:**
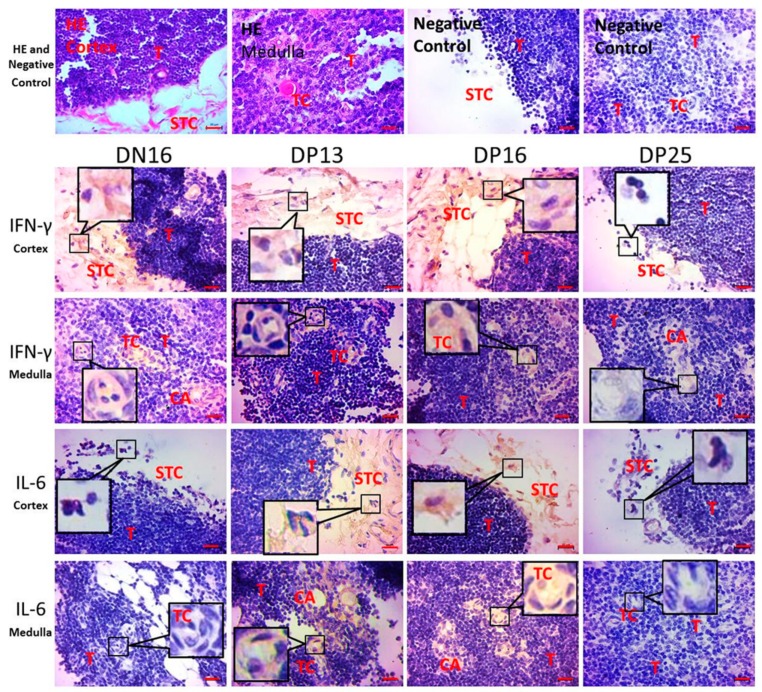
Immunohistochemical localization of interferon-gamma (IFN-γ) and interleukin (IL)-6 proteins in the thymuses. Thymus is divided into the cortex and medulla. Note: HE = stained by hematoxylin and eosin; DN16 = Day 16 of nonpregnancy; DP13 = Day 13 of gestation; DP16 = Day 16 of gestation; DP25 = Day 25 of gestation; T = Thymocyte; STC = Stromal cell; CA = Capillary; TC = Thymic corpuscles. Bar = 20 µm.

The staining intensity for IFN-γ was strong in the thymic cortex on day 16 of nonpregnancy, days 13 and 16 of gestation, and there was almost no staining on day 25 of pregnancy in the thymic cortex (Figure 3). The staining intensity for IL-6 was strong on days 13 and 16 of gestation, and there was almost no staining in the thymic cortex on day 16 of nonpregnancy and day 25 of gestation (Figure 3). Furthermore, IFN-γ and IL-6 proteins were located in the stromal cells in the cortex, capillaries, and thymic corpuscles in the medulla (Figure 3).

## 4. Discussion

The maternal immune rejection to the semi-allogeneic fetus is suppressed during gestation in domestic ruminants [17,18]. Thymus is a main organ of the immune system, and immunologic function of maternal thymus is modulated during gestation in mice and humans [19,20]. It is mediated by hormones [10] that the maternal thymus undergoes significant involution during pregnancy in mammals [9]. It is known that Th cytokines are implicated in maternal immune regulation during pregnancy. The primary pregnancy recognition signal (IFNT) secreted by the conceptus is detected between days 14 and 21 of pregnancy, and induces changing expression of interferon stimulated genes in peripheral blood leukocytes [21], CL [22,23], bone marrow [24], and thymus [12] through blood circulation in sheep. In the present study, early pregnancy influenced the expression of Th1 and Th2 cytokines in the maternal thymus, which indicated that the maternal thymus participated in the systemic immunoregulation during early pregnancy in sheep.

IFN-γ is mainly produced by Th1 cells, and participates in immunostimulation and immunomodulation [25]. IFNT is an immunomodulatory cytokine that enhances IFN-γ responses by effector T cells in vitro, which are not strictly Thl-biased responses in cattle [26]. However, there is a significant increase in the IFN-γ levels in sera from pregnant goats infected with *Neospora caninum* from day 40 to 90 of gestation, when abortion occurs [27]. We previously found that value of *IFN-γ* mRNA is downregulated in bovine PBMCs on days 18 and 30 of gestation [7]. In this study, there was a peak in the value of IFN-γ on day 16 of pregnancy (Figure 1 and Figure 2), which may be induced by IFNT through blood circulation. Furthermore, the level of *IFN-γ* mRNA and protein declined from day 16 to 25 of pregnancy, which may be due to lack of IFNT stimulus, and was needed for successful pregnancy in sheep.

TNF-β (lymphotoxin-α), is a member of the tumor necrosis factor family produced by lymphocytes, and is involved in the development of peripheral lymphoid organs [28]. TNF-β participates in regulation of immune cells, and is related to poor fetal growth among European Americans [29]. It has been reported that TNF-β-dependent pathway modulates the thymic emigration of Valpha14 invariant natural killer (NK) T cells (a distinct immunoregulatory T cell lineage) from the thymus to the periphery in mice, and thymic emigration of T cells is a necessary step in immunity [30]. In this study, there was an increase in the values of TNF-β (Figure 1 and Figure 2), which suggested that TNF-β was implicated in the regulation of thymic immunity during pregnancy in sheep.

IL-2 is a multipotent cytokine, promotes or inhibits cytokine cascades that relate to T helper cell differentiation state through modulating critical expression of the receptors of other capital cytokines and transcription factors, and plays important roles in regulation of immune functions through enhancing the development of T cells [31]. It has been reported that IL-2 exerts its effects on the immune response via enhancing naive CD4^+^ T cell differentiation into T helper cells [32]. IL-2 exerts a significant stimulatory effect on prostaglandin E2 (PGE2) release from chorion tissue through the cyclooxygenase pathway in women [33]. PGE2 is implicated in the prevention of luteolysis, which is helpful for pregnancy maintenance. However, it is reported that there is a similar serum concentration of IL-2 in anoestrus, dioestrus, and second week of pregnancy in bitches [34]. Our results indicated that ovine early pregnancy has no significant effects on IL-2 expression in the thymuses (Figure 1 and Figure 2), which indicated that IL-2 was not crucial for the maternal thymic immunomodulation.

IL-4 promotes Th2 immunity via suppressing Th1 immunity, and there is an increase in the IL-4 level throughout normal pregnancy [35]. However, it has been reported that there are successful gestations with fetus growth and development normally in IL-4-knockout mouse, which suggests that IL-4 is not crucial for the successful pregnancy [36]. We found that relative values of IL-4 were almost identical in nonpregnant and pregnant animals (Figure 1 and Figure 2). It is through PGR and an immunomodulatory protein (PIBF) that P4 promotes the secretion of IL-4 [37]. We reported recently that early gestation has no significant effects on expression of the PGR isoform (89 kDa) and PIBF variant (80 kDa) in ovine thymus [12], which may result in the similar expression of IL-4. It is also reported that successful pregnancy is not related with IL-4, and IL-4-dependent Th2-type responses are not vital for maternal tolerance in mice [34], which is similar to the results in the present study. Therefore, we suggested that IL-4 was not crucial in ovine thymus during early gestation.

IL-5 is a growth factor and a Th2 cytokine, which induces B cell growth to secret antibody. Spontaneous secretion of IL-5 is lower in cell culture supernatants from PBMCs in preeclampsia than that in successful gestation, which manifests that systemic Th2 immunity of preeclamptic women is downregulated [38]. Placental growth factor upregulates IL-5 production of the CD4^+^ T cells, which is more beneficial for successful gestation in women [39]. During pregnancy in women, pregnancy-specific glycoprotein releases into blood circulation, promotes the development of IL-5-secreting cells to protect against *Listeria monocytogenes* infection, which is implicated in the successful pregnancy [40]. Our results indicated that the higher value of IL-5 was present on days 16 and 25 of gestation than that in other two pregnant statuses (Figure 1 and Figure 2). Therefore, upregulated expression of IL-5 may be necessary for pregnancy maintenance in sheep.

IL-6 has both pro- and anti-inflammatory properties, and participates in activation of the immune system. There is an increase in the endometrial *IL-6* mRNA, and IL-6 is a vital component of embryo-uterine interactions, and helpful for normal conceptus implantation in early pregnant pigs [41]. The extravillous and cytotrophoblast produce IL-6, and IL-6 is implicated in regulating the invasiveness of embryonic ectoderm cells and the functions of extravillous and cytotrophoblast, which is essential for successful placentation [42,43]. Our data revealed that IL-6 value was significantly high on days 13 and 16 of pregnancy (Figure 1 and Figure 2), which demonstrated that IL-6 may contribute to the invasiveness of embryonic ectoderm cells through endocrine manner in sheep. However, there was a decline on day 25 of gestation (Figure 1 and Figure 2). It is reported that IL-6 level of maternal plasma is elevated in preterm delivery women during gestation [44], and high serum IL-6 level is associated with pregnancy-induced hypertension in pregnant women [45], which is consistent with our results that there is a decline of IL-6 on day 25 of gestation in ewes.

As a multiple, pleiotropic cytokine, IL-10 decreases Th1 cytokines expression in macrophages. Dendritic cell (DC) and NK cell interactions influence the development of maternal tolerance during early gestation. IL-10 produced by uterine NK cells is necessary to regulate DC phenotype and pregnancy success [46]. Immune and nonimmune cells produce IL-10 that is an immune suppressive factor. IL-10 has vital effects on maintaining immune tolerance, and also serves as an immunotherapeutic intervention in treatment of adverse pregnant outcomes [47]. It has been reported that exogenous IL-10 administration can prevent preeclampsia-like symptoms through regulating immune cell subsets related with adaptive response during pregnancy in mice [48]. The present data revealed that the values of IL-10 in pregnant groups were higher than that in nonpregnant group (Figure 1 and Figure 2), which was similar to the above reports, and proposed that upregulation of IL-10 is essential for successful gestation in sheep.

Thymus is a specialized organ of immune system, participates in T cell maturation, and is comprised of medulla and cortex. There are lymphocytes and cortical thymic epithelial cells in thymic cortex, while concentric squamous cells, characteristic whorled bodies called Hassall corpuscles or thymic corpuscles in thymic medulla, and fewer lymphoid cells and coarser network of reticular than that in the cortex. The thymus is involved in T-cell generation and maturation, which is essential for regulation of adaptive immune response. It is through steroid hormones that endocrinology meets immunology in thymus [49]. Intrathymic processes are necessary for the maturation of gamma delta T cells that play a vital role in the maintenance of pregnancy [50,51]. Our immunohistochemistry data revealed that the immunostained-IFN-γ and -IL-6 proteins were located in the stromal cells in the cortex, capillaries, and thymic corpuscles in the medulla (Figure 3). Thymic cortex exhibits a positive selective activity for thymocyte, while medulla is involved in negative selection for thymocyte, which allows continuing thymocyte development. The blood circulates to the thymus via capillary, and early pregnancy regulated expression of IFN-γ and IL-6 in specific thymic cells through blood circulation. Therefore, it was speculated that early pregnancy affected the functions of epithelial reticular cells, capillaries, and thymic corpuscles, which regulated the thymic T cell development in the cortex and medulla.

## 5. Conclusions

There was a downregulation of IFN-γ after an increase on day 16 of pregnancy, a peak in the expression of IL-6 on day 13 of pregnancy, and TNF-β, IL-5, and IL-10 upregulated in the thymuses during early gestation. In addition, IFN-γ and IL-6 proteins were located in the stromal cells, capillaries, and thymic corpuscles. Therefore, it was speculated that early gestation exerted its effects on the thymus to modulate the secretion of Th cytokines, which was necessary for successful pregnancy in sheep.

## Figures and Tables

**Table 1:** Primers used for qRT-PCR of ovine helper T (Th)1 and Th2 cytokines.

Gene	Primer	Sequence	Size (bp)
*IL-2*	Forward	AAACCTGAACACCAGAGAGAT	117
	Reverse	GCCTTTACTGTCGCATCA	
*IFN-γ*	Forward	TTGAACGGCAGCTCTGAGAA	124
	Reverse	TTGGCGACAGGTCATTCATC	
*TNF-β*	Forward	CCACTGACGGGCTTTACCT	141
	Reverse	TGATGGCAGAGAGGATGTTG	
*IL-4*	Forward	CCAAAGAACGCAACTGAGAA	120
	Reverse	GCTGCTGAGATTCCTGTCAA	
*IL-5*	Forward	CATCTGCGTTTGACCTTGG	139
	Reverse	AGTTCCCATCACCTATCAGCA	
*IL-6*	Forward	CGAGTTTGAGGGAAATCAGG	118
	Reverse	GTCAGTGTGTGTGGCTGGAG	
*IL-10*	Forward	CTCTGTTGCCTGGTCTTCCT	169
	Reverse	TGTTCAGTTGGTCCTTCATTTG	
*GAPDH*	Forward	GGGTCATCATCTCTGCACCT	176
	Reverse	GGTCATAAGTCCCTCCACGA	
